# Delivering Assessment and Treatment at Scale in Mental Health–Translation from Low-Resource Settings

**DOI:** 10.1192/bjo.2026.11091

**Published:** 2026-06-30

**Authors:** Ethan Lowden, Ruchika Gajwani, Helen Minnis

**Affiliations:** University of Glasgow, Glasgow, United Kingdom

## Abstract

**Aims:**

Mental health problems are the leading contributors to disease in children, with rising prevalence exerting significant pressures on Child and Adolescent Mental Health Services (CAMHS). Services are currently unable to meet demand, resulting in prolonged waiting lists.

CAMHS operates on a crisis-driven model, despite the recent WHO and Darzi reports recommending early intervention strategies, including the use of community-based lay practitioners to do initial assessments.

The aim in this study was to explore the translation of community-based interventions from low-resource settings into a UK context.

**Methods:**

Participants were purposively selected from multi-agency teams and professionals working with children were invited to take part in a qualitative study using thematic analysis following informed consent. These professionals included CAMHS clinicians, managers, people with lived experience and educational professionals.

**Results:**

Six themes with four associated subthemes emerged: ‘*CAMHS Structure and Accessibility*’, ‘*Pathologisation of Suffering*’, ‘*Workforce and Professional Challenges*’, ‘*Community-based Interventions*’, ‘*Structural and Policy Reform*’, and ‘*Future Governance of Layworkers*’.

Findings emphasised the urgent need for structural and policy reform, with focus on innovative approaches to early intervention and adaptation of successful programmes from low-resource settings as models of task-sharing. This would fulfil two of the Darzi recommendations: a move from treatment to prevention, and a move from hospital to community.

**Conclusion:**

Translation of community and lay practitioner models (CLPs) into the UK has potential to improve access, promote early intervention, and align with government recommendations. However, further research using focus groups and clinical trials is required to assess the feasibility of implementing CLP training and supervision in the UK.

